# Editorial Brevities

**Published:** 1888-11

**Authors:** 


### EDITORIALS AND MISCELLANEOUS.

### EDITORIAL BREVITIES.

SVAPNIA.—We invite attention to the advertisement of this preparation as appears in this number of our journal.

DR. VAN ESMARCH, the distinguished Professor of Surgery, at the University of Kiel, is reported dangerously sick in New York City.

TRANSACTIONS of the American Association of Obstetricians and Gynecologists at the first annual meeting, held in Washington, D. C., September 18, 19 and 20, 1888.

PARKE, DAVIS & Co.—See their new advertisement on outside cover page entitled, *A new and Invaluable Vehicle for Administering Unpalatable Remedies.* The profession will thank Messrs Parke, Davis & Co., for this useful vehicle for concealing the taste of a disagreeable remedies.

PROF. A G. HOBBS.—We are pleased to note that A. G. Hobbs, M. D. Professor of Eye, Ear and Throat in the Southern Medical College, Atlanta, was elected President of the American Rhinological Association at its late meeting in Cincinnati. The next meeting is appointed for Chicago in September, 1889.

SKIN GRAFTING from the cadaver having been recently practiced and claimed as a new idea by a writer in the *Medical and Surgical Reporter* is referred to by Dr. Richard J. Levis as an "old discarded practice," he having tried it from amputated limbs many years ago, and condemned it as subjecting the patient to serious risk of specific infection.

THE HOAGLAND LABORATORY.—This Laboratory has been recently established in Brooklyn, N. Y., with a view to the study of Bacteriology. For this purpose private labororaties have been provided, and arrangements are now being made for the purchase of a library which shall contain all the literature necessary for reference in the departments of Bacteriology, Physiology and Pathology.

RECEIPTED—1888—Drs. Z. D. Emerson, E. A. Speed, J. H. Young, John A. Daniel, T. F. Bivins, R. D. Jackson, J. T. Kirkprtrick, Jeff Wilcox, M. E. Demaret, A. T. Park, H. A. Mobley, to March; G. E. Camp, to July; G W. Earle, to August; W. B. Maxwell, to September; W. W. Fills, to April; C. W. Killer, 1888; L. H. Cartledge, 1888.

McINTOSH BATTERY AND OPTICAL Co.—See the new advertisement of the above excellent house. We feel safe in recommending them as true and reliable men, and to say of their goods that we know of none better in the line of Galvanic and Faradic Batteries. Also in the line of Optical goods they stand high—as of Solar Mircoscopes and Stereopticons, Clinical Microscopes, Thermometers, Barometers, Eye Glasses, etc., etc. Their Natural Uterine Supporters have acquired a deservedly high reputation.

